# Analysis of Dengue and SARS-CoV-2 Coinfection in a Tertiary Care Hospital: A Retrospective Study

**DOI:** 10.1155/2024/6788850

**Published:** 2024-09-19

**Authors:** Vinayaka Korishetty, Pooja Rao, Suchitra Shenoy, Udayalaxmi Jeppu, Keerthiraj B.

**Affiliations:** Kasturba Medical College Mangalore Manipal Academy of Higher Education, Manipal, 576104, Karnataka, India

## Abstract

**Introduction:**

Coinfection of dengue virus and SARS-CoV-2 infections in dengue-endemic areas is a significant public health concern. Coinfections can result in severe illness. Hence, this study determines the incidence of dengue and COVID-19 coinfection for a better understanding of the clinical presentation, laboratory parameters, and outcomes including mortality.

**Methods:**

The patients admitted to two tertiary hospitals with RT PCR-proven COVID-19 infection and dengue positive by NS1 rapid antigen or IgM dengue ELISA for two years between January 2020 and December 2022 were considered. Clinical data were retrieved from medical records including the laboratory findings and outcomes of these patients. The categorical data were analyzed in the form of frequency and proportion. The quantitative data were analyzed in the form of mean, median, and proportion.

**Results:**

Out of 2301 confirmed dengue samples and 3718 confirmed COVID-19 samples, there were 14 cases of coinfection with the presence of COVID-19 and dengue infection at the same time. ICU admission of 14.2% and mean hospital stay of 7 days were noted. Mainly the symptoms reported were fever at 92.9%, myalgia at 35.7%, and headache, vomiting, and cough at 28.6%. The laboratory findings were elevated lactate dehydrogenase and C-reactive protein in 100% of patients, elevated ferritin in 92.9%, thrombocytopenia in 71.4%, elevated AST and ALT in 71.4%, and elevated D-dimer in 57.1% of patients. There was no effect on morbidity and mortality seen among coinfection.

**Conclusion:**

COVID-19 and dengue share similar clinical features and laboratory findings. Diagnosis of one disease cannot rule out the presence of other infections. There might be chances of misdiagnosis or missed diagnosis. Hence, it is important to stress about early detection using specific methods and confirmation of disease with timely management, as it is a potentially new dimension for public health concern and management.

## 1. Introduction

Dengue is the most occurring disease throughout the tropics, which is influenced by rainfall, temperature, and urbanization, as it creates favourable conditions for breeding of *Aedes* mosquitoes. It has 4 serotypes that are known to infect humans. There are more than 100 countries where dengue is endemic, such as South American countries, Southeast Asian countries, and specific western regions. In India, dengue is the foremost public health concern, present in almost all the states. In 2016, there were more than 1,00,000 laboratory-confirmed cases in India [1]. The National Vector-Borne Disease Control Program (NVBDCP) reported 1,57,315 cases and 166 deaths in 2019 and 44,585 cases and 56 deaths in 2020 [2].

There were an estimated 96 million dengue infections globally in 2010, which contributes 70% to Asia and 34% to India alone [3]. WHO classification 2009 classified it into two stages: dengue with or without danger signs like fever, nausea, vomiting, aches and pains, diarrhea, leukopenia, abdominal pain, lethargy, restlessness, and hepatomegaly and severe dengue with plasma leakage, fluid loss, respiratory distress, dehydration, and other organ involvement [4].

SARS-CoV-2 causes COVID-19 which was first identified in December 2019 and epidemiologically linked to a local seafood market, which led to an increase in the number of cases in China and later spread worldwide resulting in WHO to declare it as a pandemic on 11 March 2020 [5, 6]. As of 16 February 2022, there were 414.5 million cases with 5.8 million deaths globally [7]. India has 42.7 million cases and 0.5 million deaths [8]. The symptoms may be fatigue, fever, anorexia, shortness of breath, myalgia, sore throat, nasal congestion, headache, nausea, vomiting, diarrhea, and loss of smell or taste [9]. Laboratory findings such as lymphopenia and elevated lymphocyte, LDH, CRP, and IL-6 and biomarkers like high procalcitonin and ferritin along with blood hypercoagulability, elevated D-dimer, and abnormality of PT and aPTT prolongation were noted [10].

During the COVID-19 pandemic, there was also an increase in dengue cases. The coinfection of these viruses has been already reported in different dengue-endemic countries like Brazil (*n* = 3), Singapore (*n* = 2), Thailand (*n* = 1), and Indonesia (*n* = 4) [11–14]. The outcome and the dynamics of each disease may be altered in the presence of infections. This study aimed to analyze the prevalence of coinfections of dengue and COVID-19 and the clinical and laboratory parameter outcomes among patients with coinfections.

## 2. Materials and Methods

An analytical, cross-sectional study was conducted for a two-year duration from March 2019 to March 2022 at KMC, Mangalore. The study proceeded after receiving the clearance from the Institutional Ethics Committee (IEC) of Kasturba Medical College, Mangalore, with IEC clearance number: IEC KMC MLR 05-2022/196. All consecutive patients admitted to the hospital with the diagnosis of COVID-19 and dengue during the study period were considered. The study agreed with the Helsinki Declaration and with the consensus of IRB to view patient's medical records. Informed consent was waived off by the ethics committee as the data were retrieved from the laboratory information system and hospital medical record department from the KMC hospitals at Attavar and Ambedkar Circle and the tests performed were a part of the investigations ordered for the patient during the diagnosis and treatment. Inclusion criteria included symptomatic patients admitted to the hospital who meet the following criteria:

1. Real-time PCR-proved SARS-CoV-2 infection
2. Dengue positive by NS1 rapid antigen test and/IgM dengue ELISA

Exclusion criteria were as follows: single infection with either COVID-19 or dengue and IgG-positive dengue by NS1 rapid antigen test.

### 2.1. Procedure

The patients with positive NS1 rapid antigen test (J Mitra Pvt Ltd, India) or IgM dengue ELISA test (Panbio) and a positive RT-PCR test for SARS-CoV-2 were considered for the study. For dengue IgM ELISA, a value of more than 11 panbio units was considered positive. Real-time PCR for COVID-19 was done for detection of SARS-CoV-2 RNA.

The clinical and laboratory parameters of these infections were noted in the patients as per the pro forma. The outcome variables in these patients such as duration of stay in the hospital, severity, and recovery/death were also noted.

The categorical data were analyzed in the form of frequency and proportion. The quantitative data were analyzed in the form of mean, median, and proportion. The data were entered and analyzed using the statistical software Statistical Package for Social Sciences (SPSS) Version 11.5.

## 3. Results


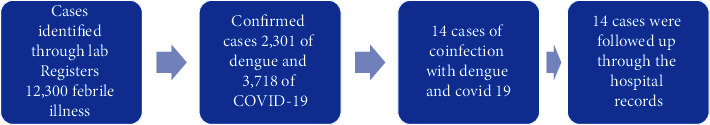


We observed that there was an equal male and female proportion (male : female (1 : 1)) in this coinfection. Most of the coinfected cases were reported in adults ranging from 18 to 59 years, with one case being pediatric, aged 7 years (Table 1). Bronchial asthma, hypertension, and diabetes were the comorbidities reported in three adult patients. Two adult patients required ICU admission, with the prevalence being 14.2% (Table 1). The mean hospital stay was 7 days. There was no effect on morbidity and mortality seen among coinfection in this study.

The rapid antigen NS1 test results were reliable in the first week of illness while IgM dengue-specific antibodies ELISA was picked after 5–7 days of infection. The diagnostic methods employed in this study included rapid antigen tests, revealing a detection rate of 78.6% for the NS1 antigen in cases of dengue and 7.1% for SARS-CoV-2. Additionally, dengue was assessed using IgM ELISA, yielding a detection rate of 64.3%, while SARS-CoV-2 viral RNA detection through RT-PCR and GeneXpert demonstrated rates of 28.6% and 100%, respectively (Table 1).

The most frequently reported symptom was fever, which was present in 92.9% of cases, and myalgia was present in 35.7% of cases followed by headache, vomiting, and cough in 28.6% of cases. Sore throat, dyspnea, and diarrhea were present in 14.3% of cases. Syncope, cold, dysphagia, abdominal pain, anorexia, edema, hematuria, and oliguria were present in 7.1% of cases (Table 1).

The most common laboratory findings were elevated LDH (100%), elevated CRP (100%), and elevated ferritin (92.9%) with the highest value of ferritin being 39,470 *μ*g/L and the mean being 6000 *μ*g/L, thrombocytopenia (71.4%) with lowest platelet count of 6000/*μ*L and mean platelet count of 1.04 lakh/*μ*L, elevated AST (71.4%) with the highest value of AST being 1716 IU/L and mean being 206 IU/L, elevated ALT (71.4%) with the highest value of ALT being 1103 IU/L and mean being 135 IU/L, elevated D-dimer (57.1%), leukopenia (50%), elevated ESR (50%), lymphopenia (50%), low hemoglobin (28.6%), high neutrophils (28.6%), high lymphocytes (21.4%), low neutrophils (14.3%), and high WBC (7.1%) (Table 1).

### 3.1. Clinical Outcomes

One patient presented with myocarditis related to dengue infection. On the day of admission, 8 patients had low platelet counts. Three patients required platelet transfusion in the hospital. All 14 patients had improved platelet counts on the day of discharge and recovered during their hospital stay. The mean days of hospital stay were 7, ranging from 4 to 12 days.

## 4. Discussion

In our study of coinfection, we desire to see the outcome of coinfected patients, in which we have not reported any mortality, which is much less than the estimated global rate for dengue and COVID-19 patients which is 1.9% and 1.05%, respectively [15, 16]. Even though it is seen that the prevalence of dengue is high among males in this study, there is an equal prevalence of males and females, with one case being pediatric [17, 18]. Though Mangalore is a dengue endemic region, the incidence of coinfections were low.

It is believed that advanced age is a major risk factor in general for all types of infections; particularly, in this type of coinfection, advanced age has more associated risk factors. There are four patients above 50 years, who did not require ICU admission, while one patient required platelet transfusion, and all 3 improved on further management. However, there were 2 patients of mid-age requiring ICU admission and longer duration hospital stays. Therefore, age cannot be a definite predictable risk factor, and also due to the limitation of sample size in this study. This study shows ICU admission of 14.2% which is significant. It also varies from population to population and healthcare facilities available in that population. This finding underscores the severity of the coinfection and emphasizes the importance of monitoring and managing these cases closely.

In one study, 87.9% of COVID-19 patients presented with fever, 67.7% presented with cough, and 13.7% presented with headache [19]. As in all types of infection, fever is one of the inclusion criteria. In another case study of dengue infection, 66.7% presented with cough which might be due to pleural effusion due to plasma leakage in dengue hemorrhagic fever [20, 21]. Coagulation mechanisms evidenced by prolonged activated partial thromboplastin time (APTT) and prothrombin time (PT) are reported to be affected in dengue illness. In another study by Imad et al., 89% of cases presented with headache and more commonly in dengue hemorrhagic fever [22]. Our study also showed fever as the most frequent symptom followed by myalgia, headache, and cough. Also, dengue infection could present with rash, and there is a case study in which COVID-19 presented with rash [23]. In our study, there was one patient who presented with rash along with respiratory symptoms. A physician cannot ignore COVID-19 infection and treat only dengue infection or vice versa, and specific tests for both need to be performed. Hematological findings are similar in COVID-19 and dengue infections like leukopenia and thrombocytopenia, and neutrophils have increased numbers due to infection which appear early in both infections, but in our study, we saw almost near normal neutrophil count. The near-normal neutrophil count observed could be reflective of a specific immunological response or variation in the host response to the coinfection or stresses the use of RT-PCR to find the definitive cause of the disease. Increased levels (72.4%) of alanine transaminase and aspartate aminotransferase were also seen in this study. In another study, it was found that AST and ALT were raised in dengue infection indicating the increase in severity of infection and common feature in viral infections [24, 25].

Many other risk factors can also contribute to COVID-19 including infection which might be due to other viral or bacterial infections [26–28]. The presence of multiple infections is life-threatening leading to the deterioration of health status, in addition to requiring ICU admission and associated complications. On the other hand, the risk of transmitting COVID-19 is due to inappropriate measures of the COVID-19 protocol which is not maintaining social distancing and not wearing masks. This region is a dengue-endemic area; most dengue cases are present throughout the year, but a rise in cases has been seen around July to December.

Dengue-endemic countries are at risk of possible coinfection and coepidemic in which dengue and COVID-19 coexist [29]. Here coinfection cases have been seen which can lead to the overlapping of clinical features and laboratory parameters making diagnosis and treatment difficult for physicians. Hence, testing methods having high sensitivity and specificity must be devised; if this coexisting infection is misdiagnosed, there would be the burden of complications, and special measures must be taken like mosquito control programs and adherence to COVID-19 protocol.

**Figure 1:**
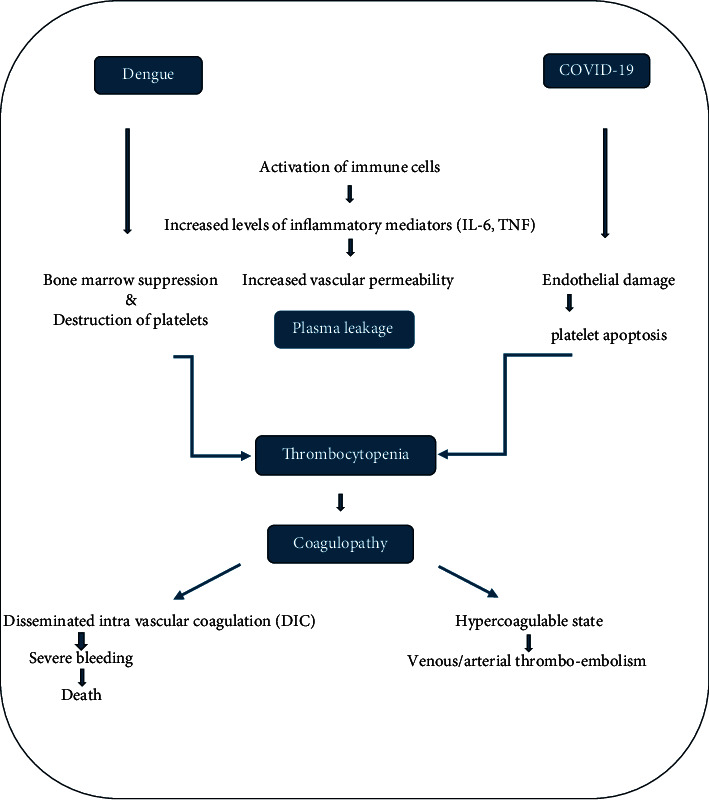
Common pathophysiological pathway followed by dengue virus and SARS-CoV-2 [30].

COVID-19 and dengue share similar pathophysiology (Figure 1) [30]. Thrombocytopenia is explained by a mechanism that results in depressed platelet synthesis due to virus-induced marrow suppression and immune-mediated clearance of platelets. Furthermore, the immune complex formed is produced, and along with the dengue virus, further destruction of platelets occurs [5, 31, 32].

Studies done in the past have shown that comorbidities have resulted in severe illness and death in both COVID-19 and dengue infections [33, 34]. Diabetes, hypertension, and digestive disease have been observed as serious comorbidities that had severe disease outcomes [14, 17, 35]. Particularly, diabetes and cardiovascular risk have been observed to be significant comorbidities for severe disease and high case fatality [36]. Therefore, patients with comorbidities should have special care and precautionary measures to avoid infection from either of the viruses.

COVID-19 disease has strong hematopoietic system symptoms and is frequently accompanied by severe blood hypercoagulability [10], which is also seen in this study by elevated levels of D-dimer, which is indicative of blood hypercoagulability. Therefore, close monitoring needs to be done in the presence of dengue infection. In severe cases, dengue infection alone leads to plasma leakage and hemoconcentration which will increase the risk of disseminated intravascular coagulation (DIC). The important point to be noted is that in this type of coinfection of SARS-CoV-2 and dengue virus, DIC is most probable to occur. Hence, early detection and thromboprophylaxis should be done as preventive measures. In our study, there is one pediatric case diagnosed with G6PD deficiency, which may be caused due to certain drugs and which is a risk factor for anemia; therefore, close monitoring of hematological parameters should be done during the treatment. In a study by Aydemir et al., it was found that COVID-19 and G6PD enhance the risk of thrombosis and hemolysis [37]. An effective intervention will enhance patient outcomes. In our study, we noticed 100% elevated levels of CRP. Recent studies have shown that the severity or development of COVID-19 is related to plasma CRP levels [38–40]. CRP is also significant in the development of dengue and has the potential to be a predictive biomarker [41, 42]. CRP is predominantly synthesized by hepatocytes in response to inflammation. In our study, we could trace only three patients' prothrombin time (PT) which was increased. Inflammation of the liver could be a point to justify this prolonged PT. In a study done by Wang et al., patients with prolonged PT at admission had considerably greater mortality rate than those with normal PT; therefore, such patients should be monitored more closely and treated with caution [43]. In dengue fever patients, APTT and PT are significantly prolonged [44]. As dengue is a part of coinfection in this study which also affects the liver, prolonged PT is also an overlapping laboratory parameter. Various studies showed that 3 patients had complications related to shock, acute respiratory distress syndrome (ARDS), and multiple organ failure. Six had fatal outcomes. The median day of stay in the hospital was 11 and ranged from 9 to 20 days [13, 14, 35].

Overall, the hematological and biochemical changes reported in SARS-CoV-2 and dengue virus coinfection individuals were linked to decreased lung function and a higher probability of hospitalization [45]. A current study shows evidence of coinfection of dengue and COVID-19 to a certain rate, which is considerable and cannot be misdiagnosed. The outcome of this study can be certain or uncertain as the sample size of coinfections was low. Limitations of the study include the sample size which can lead to biased results that may not accurately reflect a broader population. The reliability of the available diagnostics tests can vary with false positives and false negatives that may result in misclassification of the two diseases. Also, geographical variability can affect the generalizability of the parameters in the study. Furthermore, studies with a good sample size would help to study the coinfection and potentially enhance the interventional and management approaches.

## 5. Conclusion

This study shows that in the period of pandemics and in this part of the country where dengue is endemic, detecting one virus does not rule out the potential of having another infection concurrently. We see the overlapping features of dengue and COVID-19 infection. This is a clinical challenge for physicians to diagnose accurate infections. Furthermore, it emphasizes the significance of an accurate and quick diagnosis. There is a requirement of testing for both dengue and COVID-19 in areas where incidence is high since each infection has a unique clinical approach. Hence, simple and affordable rapid testing kits capable of differentiating SARS-CoV-2 and dengue virus with high sensitivity are required. In addition, to establish a specific diagnosis, additional laboratory tests have to be performed as RT PCR for detecting SARS-CoV-2 and IgM antibody detection by ELISA or viral RNA by RT PCR for detecting dengue virus are important. Therefore, a specific diagnostic test is the need of the hour for accurate and timely management of such cases. As a part of prevention, vector control in the dengue-endemic area had come to a halt due to the lockdown imposed in the country. Hence, there should be appropriate tracking of the vector control program and its activities and monitoring of the COVID-19 protocol. The current syndemic scenario that various countries are experiencing is not limited to COVID-19 and dengue fever, and it encourages governments and health professionals to improve research to support control measures and policies to combat dengue and other epidemics while implementing COVID-19 pandemic prevention measures.

## Figures and Tables

**Table 1:** Clinical and laboratory characteristics seen in dengue and COVID-19 coinfections.

	No. of patients (%)
*Age group*	
0–10	1 (7)
11–20	2 (14)
21–30	1 (7)
31–40	4 (28)
41–50	2 (14)
51–60	4 (28)

*Laboratory findings*	
High LDH, CRP	14 (100)
High ferritin	13 (92)
Low platelets, high AST, ALT	10 (71)
High D-dimer	8 (57)
Low WBCs, lymphocytes, high ESR	7 (50)
Low Hb, high neutrophils	4 (28)
High lymphocytes	3 (21)
Low neutrophils	2 (14)
High WBCs	1 (7)
Platelets transfused	3 (21)

*Testing methods employed*	
NS1 antigen	11 (78)
Rapid antigen SARS-CoV-2	1 (7)
IgM dengue ELISA	9 (64)
RT PCR SARS-CoV-2	4 (28)
GeneXpert SARS-CoV-2	14 (100)

*Symptom characteristics*	
Fever	13 (93)
Myalgia	5 (35)
Cough, headache, vomiting	4 (28)
Sore throat, diarrhea, dyspnea	2 (14)
Rash, cold, dysphagia, syncope, abdominal pain, anorexia, hematuria, oliguria, edema	1 (7)
Dehydration, ageusia, arthralgia, anosmia	0
ICU admission	2 (14)
Did not require ICU admission	12 (85)
Length of stay *n* ≤ 5 days	5 (35)
Length of stay *n* ≥ 6 days	9 (64)
Recovery on therapy	14 (100)

## Data Availability

All the data related to this study are available from the corresponding authors upon request.
